# Supplementary material for: Genome-wide identification and functional characterization of CDPK gene family reveal their involvement in response to drought stress in Gossypium barbadense
Source: PeerJ. 2022 Feb 8;10:e12883. doi: 10.7717/peerj.12883 (PMC8833227; doi:10.7717/peerj.12883)
Supplement: Appendix S1A [file peerj-10-12883-s001.docx]

RT-PCR and qPCR reaction systems and procedures

1. RT-PCR amplification reaction system

| Component | Volume (μl) |
| --- | --- |
| Forward Primer | 0.5 |
| Reverse Primer | 0.5 |
| Template | 1 |
| Mix | 8 |
| ddH_2_O | to 20 |

1. RT-PCR amplification program

| No. | Temperature (℃) | Time | Cycles |
| --- | --- | --- | --- |
| 1 | 94 | 4min | 1 |
| 2 | 94 | 30s | 35 |
| 3 | 59 | 30s |  |
| 4 | 72 | 30s |  |
| 5 | 72 | 7min | 1 |
| Save | 16 | ∞ |  |

1. qPCR reaction system

| Component | Volume (μl) |
| --- | --- |
| Forward Primer | 0.25 |
| Reverse Primer | 0.25 |
| Template | 0.5 |
| 2×qPCR SYBR Green Master Mix | 5 |
| ddH_2_O | to 10 |

1. qPCR amplification program

| No. | Temperature (℃) | Time | Cycles |
| --- | --- | --- | --- |
| 1 | 95 | 5min | 1 |
| 2 | 95 | 20s | 40 |
| 3 | 60 | 20s |  |
| 4 | 72 | 20s |  |
| 5 | 60 | 60s | 1 |
| 6 | 95 | 15s | 1 |
