# Supplementary material for: Genome-wide identification and functional characterization of CDPK gene family reveal their involvement in response to drought stress in Gossypium barbadense
Source: PeerJ. 2022 Feb 8;10:e12883. doi: 10.7717/peerj.12883 (PMC8833227; doi:10.7717/peerj.12883)
Supplement: Appendix S2B [file peerj-10-12883-s002.docx]

Primers used in the experiment

1. qRT-PCR Primers

| Primer name | Primer sequence（5’-3’） |
| --- | --- |
| GbCDPK4 qRT-F | GACGACAGCAGCAACCAAAG |
| GbCDPK4 qRT-R | CTCCCTCCAAACATCGTCGT |
| GbCDPK19 qRT-F | GAATGAACGGCTTACGGCTG |
| GbCDPK19 qRT-R | GAGCCACTCTCATCCGTGTC |
| GbCDPK23 qRT-F | GCAGGCTGCGGACGTAAATA |
| GbCDPK23 qRT-R | TGTAACCACTGCCGTCTCTG |
| GbCDPK32 qRT-F | CACTGAGTCACCGAACACGA |
| GbCDPK32 qRT-R | TGCCCCCTTGCTACTATCCT |
| GbCDPK44 qRT-F | TGCGTGAAGTCGATCAGGAC |
| GbCDPK44 qRT-R | TTACCCTCACGACCCCTCTT |
| GbCDPK66 qRT-F | CTGTTGGCAGCCTTCTCGTA |
| GbCDPK66 qRT-R | GCCTTCCTGCATCATGGCTA |
| GbCDPK68 qRT-F | CTTGAGGAGCTTCGGATGGG |
| GbCDPK68 qRT-R | GCGCCCATCCTTGTCAGTAT |
| GbCDPK74 qRT-F | CCGAACTCGAATTGGGGACA |
| GbCDPK74 qRT-R | TCTGCTATCGTCTTCGCCAC |
| GbCDPK78 qRT-F | GAGGGCATTACACCGAGAGG |
| GbCDPK78 qRT-R | TACGGGCTTCCAACCACATC |
| GbCDPK80 qRT-F | TATGAGGATGCAATGGCGGT |
| GbCDPK80 qRT-R | AGCGAATCCTCTTGCTGGTT |
| GbCDPK83 qRT-F | ACACTGACAACAGTGGCACA |
| GbCDPK83 qRT-R | CATGTCACGGAACTCCTCGT |
| GhUBQ7 qRT-F | GCACCTTAGCCGACTACAAC |
| GhUBQ7 qRT-R | CATTCCGCATTAGGGCACTC |

1. VIGS vector primer

| Primer name | Primer sequence（5’-3’） |
| --- | --- |
| GbCDPK32vigsF | GGAATTCTTCTGAAATCGTGGGGAGTC |
| GbCDPK32vigsR | GGGGTACCAACCAGGGATGTTCAAGCAC |
| GbCDPK68vigsF | GCTCTAGAGATTGTTGCAAGGGGACACT |
| GbCDPK68vigsR | GGGGTACCTGTTCAAGCACTTCCTGTGC |
| GbCDPK74vigsF | GGAATTCATCCCACTGAGTCACCGAAC |
| GbCDPK74vigsR | GGGGTACCCAATTCCCCTCCTTCACAAA |
| GbCDPK80vigsF | CGGGATCCTGATGTCTGGAGTGCAGGAG |
| GbCDPK80vigsR | CCGCTCGAGTTGACCACTGTTGTCCGTGT |
| GbCDPK83vigsF | CGGGATCCTTCAACCCCAGGTCTACCAC |
| GbCDPK83vigsR | CCGCTCGAGAACAATTCACCACCAGCACA |
