# Supplementary material for: Genome-wide identification and functional characterization of CDPK gene family reveal their involvement in response to drought stress in Gossypium barbadense
Source: PeerJ. 2022 Feb 8;10:e12883. doi: 10.7717/peerj.12883 (PMC8833227; doi:10.7717/peerj.12883)
Supplement: Table S1 [file peerj-10-12883-s003.docx]

Tab 1 Molecular structure physiological and biochemical characteristics of CDPK family in *G.barbadense*

| Gene ID | Name | Start | End | Strand | CDS  (bp) | No.of aa | MW (kDa) | PI | GRANY | No.of EFh | N-myristoylation | Palm | Subcellular localization |
| --- | --- | --- | --- | --- | --- | --- | --- | --- | --- | --- | --- | --- | --- |
| GB_A01G0767 | GbCDPK1 | 11582535 | 11585598 | + | 1614 | 537 | 60.465 | 6.47 | -0.491 | 4 | yes | yes | chlo |
| GB_A01G1403 | GbCDPK2 | 45977791 | 45980529 | + | 1806 | 601 | 67.667 | 5.319 | -0.7 | 4 | yes | yes | nucl |
| GB_A02G0164 | GbCDPK3 | 1555610 | 1559769 | - | 1599 | 532 | 60.713 | 5.709 | -0.404 | 4 | yes | yes | chlo |
| GB_A02G0640 | GbCDPK4 | 9249955 | 9252681 | + | 1536 | 511 | 57.143 | 5.508 | -0.276 | 4 | No | yes | chlo |
| GB_A02G1177 | GbCDPK5 | 47660631 | 47663873 | + | 1560 | 519 | 58.613 | 5.897 | -0.462 | 4 | No | yes | chlo |
| GB_A02G1323 | GbCDPK6 | 69167338 | 69171519 | + | 1596 | 531 | 60.226 | 6.154 | -0.524 | 4 | yes | yes | cyto |
| GB_A02G1996 | GbCDPK7 | 101513161 | 101517960 | + | 1626 | 541 | 61.306 | 9.456 | -0.562 | 4 | yes | no | cyto |
| GB_A03G2018 | GbCDPK8 | 102612332 | 102615363 | - | 1707 | 568 | 63.568 | 6.118 | -0.408 | 4 | no | yes | nucl |
| GB_A04G0174 | GbCDPK9 | 2508807 | 2511925 | - | 1575 | 524 | 58.824 | 6.251 | -0.524 | 4 | yes | yes | cyto |
| GB_A04G0603 | GbCDPK10 | 16835097 | 16838821 | + | 1947 | 648 | 71.648 | 5.128 | -0.528 | 4 | no | yes | extr |
| GB_A04G0604 | GbCDPK11 | 16864839 | 16867630 | + | 1014 | 337 | 38.102 | 4.313 | -0.389 | 4 | no | no | cyto |
| GB_A04G0612 | GbCDPK12 | 17379902 | 17385337 | - | 1719 | 572 | 63.54 | 5.16 | -0.418 | 4 | no | yes | chlo |
| GB_A04G1150 | GbCDPK13 | 70886210 | 70890970 | - | 1584 | 527 | 59.394 | 6.393 | -0.405 | 4 | no | yes | cyto |
| GB_A04G1388 | GbCDPK14 | 77495936 | 77497606 | - | 912 | 303 | 34.34 | 4.468 | -0.251 | 4 | no | no | cysk |
| GB_A05G0759 | GbCDPK15 | 7051390 | 7053708 | + | 1599 | 532 | 59.519 | 5.314 | -0.578 | 4 | yes | yes | cyto |
| GB_A05G1856 | GbCDPK16 | 17672902 | 17676345 | - | 1554 | 517 | 58.274 | 5.76 | -0.496 | 4 | no | yes | cyto |
| GB_A05G2804 | GbCDPK17 | 31062713 | 31067607 | + | 1509 | 502 | 56.467 | 5.531 | -0.287 | 4 | no | yes | chlo |
| GB_A05G3356 | GbCDPK18 | 62198484 | 62200680 | + | 1605 | 534 | 60.699 | 7.095 | -0.373 | 4 | yes | yes | mito |
| GB_A06G0011 | GbCDPK19 | 85800 | 88641 | - | 1581 | 526 | 59.226 | 6.089 | -0.447 | 4 | yes | yes | vacu |
| GB_A06G2365 | GbCDPK20 | 117779296 | 117781450 | + | 1584 | 527 | 59.342 | 5.082 | -0.424 | 4 | yes | yes | chlo |
| GB_A07G1310 | GbCDPK21 | 23767798 | 23770149 | - | 1542 | 513 | 57.261 | 5.144 | -0.526 | 4 | yes | yes | cyto |
| GB_A07G1337 | GbCDPK22 | 24482137 | 24484693 | - | 1479 | 492 | 55.348 | 6.921 | -0.262 | 2 | no | yes | cyto |
| GB_A08G0136 | GbCDPK23 | 1093447 | 1096595 | - | 1617 | 538 | 60.48 | 5.241 | -0.512 | 4 | yes | yes | cyto |
| GB_A09G1375 | GbCDPK24 | 63045982 | 63048337 | - | 1572 | 523 | 58.525 | 5.527 | -0.469 | 4 | yes | yes | cyto |
| GB_A09G1415 | GbCDPK25 | 63739562 | 63742974 | - | 1596 | 531 | 59.857 | 6.583 | -0.42 | 3 | no | yes | cyto |
| GB_A09G1515 | GbCDPK26 | 65399066 | 65401968 | - | 1596 | 531 | 60.216 | 7.03 | -0.513 | 3 | no | yes | cyto |
| GB_A09G1609 | GbCDPK27 | 66869098 | 66872407 | - | 1833 | 610 | 68.228 | 4.844 | -0.502 | 4 | no | yes | nucl |
| GB_A10G0127 | GbCDPK28 | 1098874 | 1102313 | - | 1659 | 552 | 62.175 | 6.725 | -0.444 | 4 | yes | yes | chlo |
| GB_A10G1024 | GbCDPK29 | 19493606 | 19497377 | + | 1635 | 544 | 61.936 | 8.628 | -0.539 | 4 | yes | yes | cyto |
| GB_A10G1539 | GbCDPK30 | 71767387 | 71772333 | + | 1614 | 537 | 61.309 | 6.145 | -0.412 | 4 | yes | yes | chlo |
| GB_A10G2305 | GbCDPK31 | 102158746 | 102163931 | - | 1737 | 578 | 64.421 | 5.081 | -0.379 | 4 | no | yes | chlo |
| GB_A11G0231 | GbCDPK32 | 2040940 | 2043866 | - | 1593 | 530 | 60.366 | 6.87 | -0.525 | 4 | no | yes | mito |
| GB_A11G0322 | GbCDPK33 | 2896532 | 2899523 | + | 1653 | 550 | 62.326 | 6.526 | -0.442 | 4 | no | yes | cyto |
| GB_A11G1844 | GbCDPK34 | 23581715 | 23586008 | - | 1665 | 554 | 62.701 | 9.481 | -0.548 | 4 | yes | no | cyto |
| GB_A12G0119 | GbCDPK35 | 1598637 | 1601369 | - | 1683 | 560 | 62.564 | 5.809 | -0.337 | 4 | no | yes | pero |
| GB_A12G2620 | GbCDPK36 | 97776054 | 97779676 | - | 1605 | 534 | 60.127 | 6.181 | -0.514 | 4 | yes | yes | cyto |
| GB_A13G0029 | GbCDPK37 | 243684 | 247052 | - | 1740 | 579 | 64.677 | 6.055 | -0.396 | 4 | no | yes | chlo |
| GB_A13G0329 | GbCDPK38 | 3614861 | 3616910 | - | 633 | 210 | 24.112 | 5.487 | -0.353 | 2 | no | no | pero |
| GB_A13G0704 | GbCDPK39 | 13878754 | 13881378 | - | 1572 | 523 | 58.515 | 6.86 | -0.163 | 3 | no | yes | chlo |
| GB_A13G0708 | GbCDPK40 | 14087591 | 14090514 | - | 1587 | 528 | 58.996 | 4.98 | -0.205 | 4 | yes | yes | cyto |
| GB_A13G1739 | GbCDPK41 | 95957702 | 95961578 | - | 1611 | 536 | 60.359 | 6.278 | -0.522 | 4 | yes | yes | cyto |
| GB_A13G2610 | GbCDPK42 | 111598915 | 111603584 | + | 1584 | 527 | 58.746 | 6.278 | -0.484 | 4 | no | yes | cyto |
| GB_D01G0807 | GbCDPK43 | 10179267 | 10182297 | + | 1614 | 537 | 60.476 | 6.565 | -0.482 | 4 | yes | yes | chlo |
| GB_D01G1483 | GbCDPK44 | 28688276 | 28690890 | + | 1776 | 591 | 66.337 | 5.181 | -0.673 | 4 | yes | yes | cyto |
| GB_D02G0173 | GbCDPK45 | 1684000 | 1687786 | - | 1599 | 532 | 60.733 | 5.356 | -0.412 | 4 | yes | yes | chlo |
| GB_D02G0685 | GbCDPK46 | 8701838 | 8704554 | + | 1527 | 508 | 56.842 | 5.505 | -0.265 | 4 | no | no | chlo |
| GB_D02G2175 | GbCDPK47 | 64579782 | 64582833 | - | 1707 | 568 | 63.542 | 6.021 | -0.401 | 4 | no | yes | nucl |
| GB_D03G0089 | GbCDPK48 | 613330 | 618090 | - | 1626 | 541 | 61.286 | 9.364 | -0.559 | 4 | yes | no | cyto |
| GB_D03G0816 | GbCDPK49 | 24673806 | 24676994 | + | 1608 | 535 | 60.638 | 6.861 | -0.525 | 3 | yes | yes | E.R |
| GB_D03G0908 | GbCDPK50 | 31999433 | 32005020 | - | 1563 | 520 | 58.722 | 6.25 | -0.47 | 4 | no | yes | nucl |
| GB_D04G1067 | GbCDPK51 | 32704067 | 32710291 | - | 1719 | 572 | 63.582 | 5.166 | -0.416 | 4 | no | yes | chlo |
| GB_D04G1072 | GbCDPK52 | 32445733 | 32449451 | + | 1947 | 648 | 71.854 | 5.131 | -0.537 | 4 | no | yes | extr |
| GB_D04G1536 | GbCDPK53 | 48173656 | 48178416 | - | 1584 | 527 | 59.443 | 6.393 | -0.402 | 3 | no | yes | cyto |
| GB_D04G1768 | GbCDPK54 | 52975035 | 52977435 | - | 1659 | 552 | 61.943 | 5.289 | -0.403 | 4 | no | yes | chlo_mito |
| GB_D05G0747 | GbCDPK55 | 6221700 | 6224005 | + | 1599 | 532 | 59.506 | 5.314 | -0.562 | 4 | yes | yes | nucl |
| GB_D05G1883 | GbCDPK56 | 16168407 | 16171851 | - | 1554 | 517 | 58.311 | 5.6 | -0.493 | 4 | no | yes | cyto |
| GB_D05G2798 | GbCDPK57 | 26726415 | 26731296 | + | 1509 | 502 | 56.401 | 5.417 | -0.277 | 4 | no | yes | chlo |
| GB_D05G3361 | GbCDPK58 | 50844460 | 50846669 | - | 1605 | 534 | 60.717 | 7.242 | -0.375 | 4 | yes | yes | mito |
| GB_D05G3960 | GbCDPK59 | 64180852 | 64183954 | + | 1575 | 524 | 58.701 | 6.467 | -0.528 | 4 | yes | yes | cyto |
| GB_D06G0010 | GbCDPK60 | 61880 | 64723 | - | 1581 | 526 | 59.098 | 6.262 | -0.436 | 4 | yes | yes | vacu |
| GB_D06G1000 | GbCDPK61 | 18019256 | 18021305 | - | 468 | 155 | 17.99 | 6.677 | -0.643 | 2 | no | no | pero |
| GB_D06G2464 | GbCDPK62 | 62424298 | 62426453 | + | 1584 | 527 | 59.332 | 5.232 | -0.428 | 4 | yes | yes | chlo |
| GB_D07G1308 | GbCDPK63 | 18200223 | 18202582 | - | 1542 | 513 | 57.312 | 5.144 | -0.545 | 4 | yes | yes | cyto |
| GB_D07G1335 | GbCDPK64 | 18826598 | 18829360 | - | 1674 | 557 | 62.47 | 7.584 | -0.336 | 2 | no | yes | cyto |
| GB_D08G0136 | GbCDPK65 | 1063319 | 1066474 | - | 1617 | 538 | 60.512 | 5.101 | -0.5 | 4 | yes | yes | cyto |
| GB_D09G1222 | GbCDPK66 | 39584877 | 39587196 | - | 1575 | 524 | 58.594 | 5.408 | -0.471 | 4 | yes | yes | nucl |
| GB_D09G1259 | GbCDPK67 | 40113006 | 40116433 | - | 1596 | 531 | 59.787 | 6.523 | -0.414 | 3 | no | yes | cyto |
| GB_D09G1351 | GbCDPK68 | 41358662 | 41361530 | - | 1596 | 531 | 60.198 | 6.756 | -0.527 | 3 | no | yes | cyto |
| GB_D09G1453 | GbCDPK69 | 42647449 | 42650750 | - | 1833 | 610 | 68.214 | 4.964 | -0.517 | 4 | no | yes | nucl |
| GB_D10G0129 | GbCDPK70 | 1044375 | 1047818 | - | 1656 | 551 | 62.015 | 6.166 | -0.482 | 4 | yes | yes | chlo |
| GB_D10G0955 | GbCDPK71 | 11443501 | 11447299 | - | 1635 | 544 | 61.932 | 8.627 | -0.547 | 4 | yes | yes | cyto |
| GB_D10G1423 | GbCDPK72 | 24303866 | 24308799 | - | 1602 | 533 | 60.798 | 5.909 | -0.414 | 4 | yes | yes | cyto |
| GB_D10G2278 | GbCDPK73 | 57203131 | 57207473 | - | 1764 | 587 | 65.36 | 5.143 | -0.391 | 4 | no | yes | chlo |
| GB_D11G0227 | GbCDPK74 | 1885840 | 1888760 | - | 1593 | 530 | 60.356 | 6.564 | -0.517 | 4 | no | yes | mito |
| GB_D11G0328 | GbCDPK75 | 2611957 | 2615004 | + | 1653 | 550 | 62.326 | 6.526 | -0.442 | 4 | no | yes | cyto |
| GB_D11G1890 | GbCDPK76 | 19890847 | 19895151 | - | 1665 | 554 | 62.747 | 9.385 | -0.547 | 4 | yes | no | cyto |
| GB_D11G3847 | GbCDPK77 | 72205370 | 72211851 | + | 1584 | 527 | 59.266 | 6.34 | -0.346 | 3 | no | yes | chlo |
| GB_D12G0131 | GbCDPK78 | 1537446 | 1540215 | - | 1683 | 560 | 62.535 | 5.698 | -0.349 | 4 | no | yes | pero |
| GB_D12G2626 | GbCDPK79 | 57052759 | 57056376 | - | 1605 | 534 | 60.266 | 6.434 | -0.513 | 4 | yes | yes | cyto |
| GB_D13G0037 | GbCDPK80 | 271132 | 274501 | + | 1740 | 579 | 64.554 | 5.846 | -0.386 | 4 | no | yes | chlo |
| GB_D13G0585 | GbCDPK81 | 7184740 | 7187366 | - | 1572 | 523 | 58.419 | 6.411 | -0.148 | 3 | no | yes | chlo |
| GB_D13G0586 | GbCDPK82 | 7210746 | 7213668 | - | 1587 | 528 | 58.948 | 5.04 | -0.199 | 4 | yes | yes | cyto |
| GB_D13G1662 | GbCDPK83 | 46926365 | 46930220 | + | 1611 | 536 | 60.424 | 6.515 | -0.537 | 4 | yes | yes | cyto |
| GB_D13G2537 | GbCDPK84 | 59566569 | 59571199 | + | 1584 | 527 | 58.792 | 6.426 | -0.496 | 4 | no | yes | cyto |
