# Supplementary material for: Genome-wide identification and functional characterization of CDPK gene family reveal their involvement in response to drought stress in Gossypium barbadense
Source: PeerJ. 2022 Feb 8;10:e12883. doi: 10.7717/peerj.12883 (PMC8833227; doi:10.7717/peerj.12883)
Supplement: Table S2 [file peerj-10-12883-s004.docx]

**Table 1.** The Ka/Ks ratios for duplicate *GbCDPK* genes

| **Paralogous Genes** | ***Ka*** | ***Ks*** | ***Ka/Ks*** | **Duplicate** | **Purifying selection** |
| --- | --- | --- | --- | --- | --- |
| GbCDPK1/43 | 0.005608626 | 0.034864665 | 0.16086849 | Segmental | yes |
| GbCDPK2/44 | 0.001802777 | 0.033766675 | 0.053389241 | Segmental | yes |
| GbCDPK3/45 | 0.007250812 | 0.051314955 | 0.141300168 | Segmental | yes |
| GbCDPK4/46 | 0.00339968 | 0.042510745 | 0.079972252 | Segmental | yes |
| GbCDPK5/50 | 0.007490699 | 0.057458818 | 0.130366395 | Segmental | yes |
| GbCDPK6/49 | 0.013008456 | 0.041553653 | 0.313052047 | Segmental | yes |
| GbCDPK7/48 | 0.003192343 | 0.060493427 | 0.052771737 | Segmental | yes |
| GbCDPK8/47 | 0.003823613 | 0.020962063 | 0.182406314 | Segmental | yes |
| GbCDPK9/59 | 0.00820521 | 0.042275341 | 0.194089744 | Segmental | yes |
| GbCDPK12/51 | 0.261288452 | 3.066580138 | 0.08520516 | Segmental | yes |
| GbCDPK13/53 | 0.002463225 | 0.028704771 | 0.085812387 | Segmental | yes |
| GbCDPK14/54 | 0.010770889 | 0.070120237 | 0.153605996 | Segmental | yes |
| GbCDPK15/55 | 0.002415947 | 0.032461423 | 0.074425183 | Segmental | yes |
| GbCDPK16/56 | 0.005846355 | 0.038892063 | 0.150322568 | Segmental | yes |
| GbCDPK17/57 | 0.001714776 | 0.030710534 | 0.05583675 | Segmental | yes |
| GbCDPK18/58 | 0.003201285 | 0.038795321 | 0.082517305 | Segmental | yes |
| GbCDPK19/60 | 0.008257154 | 0.049592788 | 0.166499077 | Segmental | yes |
| GbCDPK20/62 | 0.009778097 | 0.048636942 | 0.201042604 | Segmental | yes |
| GbCDPK21/63 | 0.005026548 | 0.030430137 | 0.165183208 | Segmental | yes |
| GbCDPK22/64 | 0.008324291 | 0.030059388 | 0.276928169 | Segmental | yes |
| GbCDPK23/65 | 0.003979317 | 0.056658824 | 0.070232961 | Segmental | yes |
| GbCDPK24/66 | 0.015387288 | 0.030766032 | 0.50013884 | Segmental | yes |
| GbCDPK25/67 | 0.002438531 | 0.040540628 | 0.060150299 | Segmental | yes |
| GbCDPK26/68 | 0.00504133 | 0.036949493 | 0.1364384 | Segmental | yes |
| GbCDPK27/69 | 0.005598346 | 0.036817208 | 0.152057879 | Segmental | yes |
| GbCDPK28/70 | 0.008237531 | 0.04658129 | 0.176842045 | Segmental | yes |
| GbCDPK29/71 | 0.011957208 | 0.031063263 | 0.384930846 | Segmental | yes |
| GbCDPK30/72 | 0.008813615 | 0.036416761 | 0.242020849 | Segmental | yes |
| GbCDPK31/73 | 0.013215369 | 0.074841104 | 0.176579027 | Segmental | yes |
| GbCDPK32/74 | 0.012334959 | 0.067181835 | 0.183605569 | Segmental | yes |
| GbCDPK33/75 | 0.003141571 | 0.030496232 | 0.103015071 | Segmental | yes |
| GbCDPK34/76 | 0.003906768 | 0.041351768 | 0.094476433 | Segmental | yes |
| GbCDPK35/78 | 0.003880741 | 0.023922294 | 0.162222773 | Segmental | yes |
| GbCDPK36/79 | 0.0067711 | 0.050339561 | 0.134508518 | Segmental | yes |
| GbCDPK37/80 | 0.010059139 | 0.067430212 | 0.149178513 | Segmental | yes |
| GbCDPK38/61 | 0.089047351 | 0.156181558 | 0.570152792 | Segmental | yes |
| GbCDPK39/81 | 0.009131901 | 0.04399013 | 0.207589775 | Segmental | yes |
| GbCDPK40/82 | 0.007402898 | 0.04631324 | 0.159844093 | Segmental | yes |
| GbCDPK42/84 | 0.003272658 | 0.044119555 | 0.074177042 | Segmental | yes |

*Ka*: non-synonymous substitution rate; *Ks*: synonymous substitution rate.
