# Supplementary material for: Genome-wide identification and functional characterization of CDPK gene family reveal their involvement in response to drought stress in Gossypium barbadense
Source: PeerJ. 2022 Feb 8;10:e12883. doi: 10.7717/peerj.12883 (PMC8833227; doi:10.7717/peerj.12883)
Supplement: Table S3 [file peerj-10-12883-s005.docx]

Tab 3 Analysis of cis-regulatory elements in the promoters of each gene

| Gene name | Motifs related to growth and development | Motifs related to stress response |
| --- | --- | --- |
| GbCDPK1 | GCN4_motif | GATT-motif, ABRE, TC-rich repeats, MRE, MBS, chs-CMA1a,  LAMP-element, GTGGC-motif, AE-box, chs-Unit1m1, ARE，Box 4, G-Box |
| GbCDPK2 | CAT-box | TC-rich repeats, GT1-motif, TCCC-motif, P-box, AT-rich element,  ATCT-motif, CGTCA-motif, HD-Zip, TCA-element, TGACG-motif,  GATA-motif, chs-CMA2a, ARE, Gap-box, TATC-box, Box 4,G-box |
| GbCDPK3 | CAT-box | G-box, TATC-box, Box 4, TCT-motif, ARE, TCA-element, MRE,  TC-rich repeats, ABRE, GT1-motif, TCCC-motif |
| GbCDPK4 | Circadian, CAT-box, TGA-element, GCN4_motif | ARE, I-box, GATA-motif, TGACG-motif, TCT-motif, chs-CMA2a,  TATC-box, Box 4, CGTCA-motif, ATCT-motif, GT1-motif, TC-rich repeats, GA-motif, chs-CMA1a |
| GbCDPK5 | HD-Zip 1, AuxRR-core | A-box, TCA-element, ABRE, CGTCA-motif, LTR, GT1-motif,  TCCC-motif, G-box, 3-AF1 binding site, GATA-motif, TGACG-motif, ARE |
| GbCDPK6 |  | Box 4, TCA-element, GATA-motif, MRE, ARE, GT1-motif, LTR |
| GbCDPK7 | GCN4_motif, TGA-element | MSA-like, TGACG-motif, TCT-motif, ARE, G-box, Box 4,  CGTCA-motif, ABRE, GT1-motif, LTR |
| GbCDPK8 | CAT-box, circadian | GT1-motif, TCCC-motif, CGTCA-motif, ABRE, TC-rich repeats,  G-Box, Box 4, ARE, TGACG-motif, GATA-motif, TCT-motif, I-box |
| GbCDPK9 | GCN4_motif, TGA-element | G-Box, Box 4, AE-box, ARE, A-box, chs-CMA1a, ATCT-motif,  ABREGT1-motif |
| GbCDPK10 | circadian, O2-site, TGA-element | TGACG-motif, ARE, AE-box, GARE-motif, G-Box, Box 4,  TCCC-motif, GT1-motif, LTR, ATCT-motif |
| GbCDPK11 | TGA-element, CAT-box | GATA-motif, TGACG-motif, TCT-motif, GARE-motif, Box 4, G-Box, GT1-motif, ABRE, CGTCA-motif |
| GbCDPK12 | Circadian, O2-site | TC-rich repeats, ABRE, CGTCA-motif, Sp1, TCA-element, Box 4,  AT1-motif, TGACG-motif, G-box |
| GbCDPK13 | AuxRR-core, HD-Zip 1, TGA-element | I-box, TCT-motif, TGACG-motif, GATA-motif, ARE, AE-box,  TATC-box, Box 4, MRE, P-box, ATCT-motif, CGTCA-motif, MBS ,GT1-motif, A-box |
| GbCDPK14 | MBSI, O2-site, CAT-box | Box II, CGTCA-motif, ABRE, GT1-motif, chs-CMA1a, I-box, Box 4  GATA-motif, TGACG-motif, TCT-motif, AE-box, G-Box |
| GbCDPK15 | MBSI, CAT-box | GATA-motif, AT1-motif, I-box, AE-box, ARE, AT-rich sequence,  MBS, G-Box, GA-motif, TC-rich repeats, MRE, GT1-motif, TCCC-motif, ABRE, TCA-element, Box 4 |
| GbCDPK16 | O2-site, GCN4_motif, TCA-element | Gap-box, ARE, I-box, TCT-motif, GATA-motif, TGACG-motif,  G-box, Box 4, ABRE, CGTCA-motif, LTR, GT1-motif, MRE,  TC-rich repeats |
| GbCDPK17 |  | WUN-motif, GATA-motif, G-Box, Box 4, ACE, ABRE, GA-motif,  TATA-box, MBS |
| GbCDPK18 |  | G-Box, Box 4, GATA-motif, TCT-motif, ARE, TCA-element, MBS, MRE, ABRE, ATCT-motif, GT1-motif |
| GbCDPK19 | O2-site | MRE, GT1-motif, LTR, P-box, ABRE, CGTCA-motif, Sp1, MBS,  GC-motif, GATA-motif, TGACG-motif, I-box, ARE, GARE-motif,  Box 4, G-Box |
| GbCDPK20 | AuxRR-core, GCN4_motif | G-Box, TATC-box, Box 4, TCT-motif, TGACG-motif, GATA-motif,  ARE, A-box, CCAAT-box, TCA-element, TC-rich repeats, MRE,  GT1-motif, ATCT-motif, ABRE, CGTCA-motif |
| GbCDPK21 |  | TCT-motif, I-box, ARE, Gap-box, Box 4, AT-rich sequence, G-Box, TC-rich repeats, GT1-motif, TCCC-motif, P-box, MBS |
| GbCDPK22 | TGA-element, GCN4_motif | GT1-motif, ABRE, TC-rich repeats, Box II, MBS, CCAAT-box,  AE-box, Gap-box, WUN-motif, Box 4, G-Box, GARE-motif |
| GbCDPK23 |  | MRE, TC-rich repeats, CGTCA-motif, ABRE, P-box, GT1-motif,  MBS, chs-CMA1a, TGACG-motif, ARE, GARE-motif, Box 4, G-Box |
| GbCDPK24 | Circadian, AACA_motif, O2-site, CAT-box | GARE-motif, Box 4, G-box, TCT-motif, TGACG-motif, ARE, MBS  MRE, GT1-motif, LTR, P-box, CGTCA-motif |
| GbCDPK25 |  | I-box, WUN-motif, GATA-motif, ARE, AAAC-motif, Box 4,  TC-rich repeats, ABRE, ATCT-motif, TCCC-motif, TCA-element,  CCAAT-box |
| GbCDPK26 | O2-site | AE-box, ARE, 3-AF1 binding site, TCT-motif, TGACG-motif, Box 4, G-Box, ABRE, CGTCA-motif, TC-rich repeats, MBS |
| GbCDPK27 | O2-site | ARE, AE-box, TGACG-motif, TATC-box, G-box, GT1-motif,  CGTCA-motif, ABRE, MRE, TCA-element, A-box |
| GbCDPK28 | HD-Zip 1, circadian | MBS, GT1-motif, LTR, CGTCA-motif, ABRE, G-Box, Box 4,  AT-rich sequence, ARE, TCT-motif, TGACG-motif, GATA-motif |
| GbCDPK29 | O2-site, GCN4_motif | Sp1, TCA-element, CAT-box, MBS, GA-motif, ABRE, CGTCA-motif, TCCC-motif, ATC-motif, G-Box, Box 4, TCT-motif, TGACG-motif |
| GbCDPK30 |  | CCAAT-box, GA-motif, GT1-motif, ABRE, G-Box, Box 4,  GATA-motif, WUN-motif, ARE |
| GbCDPK31 |  | TGACG-motif, ARE, G-Box, Box 4, GT1-motif, LTR, P-box, ABRE, CGTCA-motif |
| GbCDPK32 |  | MBS, chs-CMA1a, LTR, TCCC-motif, GT1-motif, ATCT-motif,  ABRE, MRE, Box 4, AAAC-motif, G-Box, AE-box, ARE |
| GbCDPK33 |  | MBS, CCAAT-box, TCA-element, LTR, GT1-motif, AT-rich element, CGTCA-motif, GARE-motif, Box 4, TGACG-motif, ARE |
| GbCDPK34 |  | GT1-motif, GA-motif, Box 4, AE-box, ARE, 3-AF1 binding site,  GATA-motif, TCT-motif |
| GbCDPK35 | CAT-box, GCN4_motif, O2-site | MBS, GT1-motif, CGTCA-motif, ABRE, ATCT-motif, ARE, G-Box,  AT-rich element, TC-rich repeats, MRE, TATC-box, GARE-motif,  TGACG-motif, GATA-motif, TCT-motif, I-box |
| GbCDPK36 | GCN4_motif, | Box 4, G-Box, ACE, ARE, TCT-motif, AT1-motif, WUN-motif, Sp1, LTR, GT1-motif, ATCT-motif, ABRE, P-box |
| GbCDPK37 |  | TATC-box, Box 4, G-Box, ARE, ABRE, ATCT-motif, MRE |
| GbCDPK38 | GCN4_motif, RY-element | TATC-box, G-Box, Box 4, GARE-motif, ARE, TCT-motif, A-box, GATA-motif, LTR, P-box, ABRE |
| GbCDPK39 | GCN4_motif, MBSI | CCAAT-box, TCA-element, MRE, GARE-motif, Box 4, ARE |
| GbCDPK40 | O2-site, AuxRR-core | ABRE, GT1-motif, TCCC-motif, MRE, TC-rich repeats, Gap-box,  ARE, AE-box, I-box, TCT-motif, G-Box, Box 4 |
| GbCDPK41 | O2-site | AE-box, ARE, AT1-moti, TGACG-motif, G-Box, Box 4, MBS,  CGTCA-motif, ABRE, GT1-motif, MRE, TCA-element, |
| GbCDPK42 | MSA-like, CAT-box | GATA-motif, AE-box, G-Box, Box 4, TC-rich repeats, GT1-motif,  ABRE, AT-rich element, A-box, CCAAT-box, MBS, TCA-element |
| GbCDPK43 | GCN4_motif | LAMP-element, MBS, CCAAT-box, chs-CMA1a, TCA-element, MRE,Box 4, TC-rich repeats, GT1-motif, ABRE, CGTCA-motif, AE-box, AT-rich sequence, G-Box, TCT-motif, TGACG-motif, chs-Unit1m1,  ARE |
| GbCDPK44 | O2-site | G-Box, Box 4, Gap-box, ARE, TGACG-motif, GATA-motif, MRE, TCT-motif, TCA-element, CGTCA-motif, ATCT-motif, TCCC-motif, GT1-motif, TC-rich repeats, P-box, |
| GbCDPK45 | circadian | ARE, I-box, TGACG-motif, TCT-motif, G-box, ATC-motif, Box 4,  CGTCA-motif, ABRE, GT1-motif, MRE |
| GbCDPK46 | GCN4_motif, circadian, RY-element | TCA-element, CAT-box, MBS, Box II, ACA-motif, CGTCA-motif,  ABRE, P-box, TCCC-motif, GT1-motif, G-Box, Box 4,  3-AF1 binding site, TGACG-motif, GATA-motif, |
| GbCDPK47 | circadian | ACE, G-Box, TATC-box, Box 4, GATA-motif, TGACG-motif,  TCT-motif, ARE, MRE, TC-rich repeats, ABRE, CGTCA-motif,  AT-rich element, TCCC-motif, GT1-motif |
| GbCDPK48 | TGA-element, GCN4_motif, HD-Zip 1 | MBS, TCA-element, TCCC-motif, GT1-motif, LTR, CGTCA-motif,  ATCT-motif, MRE, Box 4, ARE, TGACG-motif |
| GbCDPK49 | TGA-element | GATA-motif, ARE, Box 4, MRE, ATCT-motif, LTR, GT1-motif,  TCA-element |
| GbCDPK50 | HD-Zip 1, O2-site, CAT-box, TGA-element, AuxRR-core | ABRE, TCCC-motif, GT1-motif, LTR, A-box, TCA-element,  GATA-motif, TCT-motif, ARE, G-Box |
| GbCDPK51 | Circadian, MSA-like, O2-site | G-Box, TGACG-motif, TCT-motif, ARE, TCA-element, Box II, LTR, CGTCA-motif, ABRE |
| GbCDPK52 | TGA-element, GCN4_motif, O2-site, circadian | CCAAT-box, CGTCA-motif, ABRE, LTR, GT1-motif, G-Box, Box 4, TATC-box, GARE-motif, AE-box, ARE, I-box, AT1-motif,  TGACG-motif |
| GbCDPK53 | HD-Zip 1, AuxRR-core, TGA-element | A-box, TCA-element, MRE, GT1-motif, P-box, ATCT-motif, ABRE, CGTCA-motif, TATC-box, Box 4, G-Box, TCT-motif, TGACG-motif, GATA-motif, I-box, ARE |
| GbCDPK54 |  | GATA-motif, ARE, Box 4, MRE, ABRE, ATCT-motif,  AT-rich element, GT1-motif, A-box, TCA-element, chs-CMA1a |
| GbCDPK55 | MBSI, TGA-element | ARE, I-box, AT1-motif, TGACG-motif, GATA-motif, Box 4, G-Box, ABRE, CGTCA-motif, GT1-motif, MRE, TC-rich repeats,  3-AF3 binding site |
| GbCDPK56 | CAT-box | Box II, MRE, TC-rich repeats, P-box, CGTCA-motif, ABRE,  TCCC-motif, LTR, TCA-element, MBS, WUN-motif, TCT-motif,  TGACG-motif, ARE, AE-box, GARE-motif, G-Box, Box 4 |
| GbCDPK57 | CAT-box, O2-site, GCN4_motif | GT1-motif, TC-rich repeats, GA-motif, ARE, I-box, Box 4, G-Box |
| GbCDPK58 | GCN4_motif, AuxRR-core | MBS, TCA-element, ABRE, Box 4, G-Box, ARE, GATA-motif |
| GbCDPK59 | GCN4_motif, MSA-like | GT1-motif, LTR, AT-rich element, ATCT-motif, ABRE, G-Box,  CGTCA-motif, CCAAT-box, TCT-motif, TGACG-motif, ARE,  GTGGC-motif, Box 4 |
| GbCDPK60 | O2-site, CAT-box | Sp1, MBS, GC-motif, MRE, LTR, GT1-motif, ATCT-motif, ABRE,  P-box, GARE-motif, Box 4, G-Box, GATA-motif, ARE |
| GbCDPK61 | O2-site | G-box, I-box, HD-Zip 3, GATA-motif, TGACG-motif, TCT-motif,  AE-box, TCA-element, MBS, CCAAT-box, ABRE, P-box, LTR |
| GbCDPK62 | GCN4_motif | ARE, TCT-motif, GATA-motif, TGACG-motif, TATC-box, Box 4,  G-Box, GT1-motif, LTR, ATCT-motif, CGTCA-motif, ABRE, MRE,  chs-CMA1a, TCA-element, A-box, Sp1 |
| GbCDPK63 | AuxRE | G-box, Box 4, GATA-motif, Gap-box, ARE, Sp1, TCA-element,  CCAAT-box, MBS, P-box, GT1-motif, TCCC-motif |
| GbCDPK64 |  | GARE-motif, G-Box, WUN-motif, TGACG-motif, Gap-box, ARE,  AE-box, LAMP-element, CCAAT-box, TC-rich repeats, CGTCA-motif, ABRE, AT-rich element, TCCC-motif, GT1-motif |
| GbCDPK65 | GCN4_motif, TGA-element | AT-rich element, ABRE, CGTCA-motif, MBS, chs-CMA1a,  AT1-motif, TGACG-motif, ARE, G-Box, Box 4, Box III |
| GbCDPK66 | CAT-box, AACA_motif, circadian | GT1-motif, CGTCA-motif, MBS, TGACG-motif, TCT-motif,  WUN-motif, AT1-motif, ARE, AE-box, GARE-motif, Box 4, G-Box |
| GbCDPK67 | CAT-box | Box 4, ARE, GATA-motif, I-box, WUN-motif, TCA-element,  TCCC-motif, MRE |
| GbCDPK68 | O2-site | G-Box, Box 4, 3-AF1 binding site, GATA-motif, TGACG-motif,  TCT-motif, AE-box, ARE, TCA-element, MBS, TC-rich repeats,  ABRE, CGTCA-motif, TCCC-motif |
| GbCDPK69 | O2-site | TGACG-motif, AE-box, ARE, GARE-motif, G-Box, TATC-box,  GA-motif, TC-rich repeats, ABRE, GT1-motif, LAMP-element,  A-box, TCA-element |
| GbCDPK70 | CAT-box, HD-Zip 1 | AE-box, ARE, TGACG-motif, TCT-motif, Box 4, G-Box, ACE, LTR, GT1-motif, ABRE, CGTCA-motif, AT-rich element, ATCT-motif,  TC-rich repeats, MRE, TCA-element |
| GbCDPK71 | circadian | Box 4, GARE-motif, ARE, TCT-motif, GATA-motif, TGACG-motif, TCA-element, CGTCA-motif, ABRE, TCCC-motif, GT1-motif, LTR, MRE, GA-motif |
| GbCDPK72 | CAT-box, TGA-element | GATA-motif, ARE, Box 4, Box III |
| GbCDPK73 | AuxRR-core | LAMP-element, MBS, GT1-motif, ATCT-motif, ABRE, P-box, Box 4  G-Box, HD-Zip 3, ARE |
| GbCDPK74 | GCN4_motif | GT1-motif, ATCT-motif, AT-rich element, ABRE, CGTCA-motif,  G-Box, Box 4, AT-rich sequence, TCT-motif, TGACG-motif, ARE |
| GbCDPK75 | O2-site | AT1-motif, TGACG-motif, chs-Unit 1 m1, ARE, GARE-motif,  G-Box, AT-rich sequence, Box 4, ABRE, CGTCA-motif, LTR,  GT1-motif, Sp1, TCA-element, GC-motif, MBS, CCAAT-box |
| GbCDPK76 | 3-AF1 binding site | Box 4, chs-CMA1a, TCA-element, TCT-motif, ARE, GT1-motif |
| GbCDPK77 |  | MRE, P-box, ABRE, ATCT-motif, MBS, ARE, GARE-motif, Box 4, G-Box |
| GbCDPK78 | O2-site | GARE-motif, G-Box, TATC-box, Box 4, TCT-motif, GATA-motif,  TGACG-motif, ARE, MBS, MRE, TC-rich repeats, ABRE,  CGTCA-motif, GT1-motif |
| GbCDPK79 | O2-site, GCN4_motif | ATCT-motif, CGTCA-motif, ABRE, LTR, GT1-motif, TCA-element,  chs-CMA1a, WUN-motif, I-box, TCT-motif, GATA-motif, Box 4,  TGACG-motif, ARE, ACE, GARE-motif, G-Box, |
| GbCDPK80 | CAT-box, AuxRR-core | MRE, TC-rich repeats, ABRE, TCCC-motif, MBS, TCT-motif,  GATA-motif, ARE, G-Box, Box 4, TATC-box |
| GbCDPK81 | RY-element | MRE, P-box, LTR, TCA-element, CCAAT-box, I-box, TCT-motif,  HD-Zip 3, Gap-box, ARE, AE-box, GARE-motif, ATC-motif, Box 4 |
| GbCDPK82 | O2-site | TCA-element, TC-rich repeats, TCCC-motif, GT1-motif, LTR, ABRE, TATC-box, Box 4, G-Box, TCT-motif, GATA-motif, chs-CMA2a,  ARE, AE-box |
| GbCDPK83 | O2-site, CAT-box | G-Box, Box 4, AT-rich sequence, TGACG-motif, ARE, AE-box,  MBS, P-box, ABRE, CGTCA-motif, GT1-motif |
| GbCDPK84 | CAT-box, RY-element | G-Box, Box 4, GATA-motif, ARE, CCAAT-box, MBS, TCA-element, TC-rich repeats, MRE, GT1-motif, AT-rich element, ABRE |
